# Supplementary material for: Comparative efficacy of eight therapeutic methods in the treatment of left main coronary artery disease: a Bayesian network meta-analysis protocol
Source: BMJ Open. 2022 Sep 6;12(9):e058886. doi: 10.1136/bmjopen-2021-058886 (PMC9453992; doi:10.1136/bmjopen-2021-058886)
Supplement: Supplementary data [file bmjopen-2021-058886supp001.pdf]

Appendix: Search Strategies

PubMed

#1"Coronary Disease"[Mesh] or "Left Main Disease*"[tw] or "Coronary Arteriosclerosis*"[tw]
#2"Coronary Artery Bypass"[Mesh] or "Myocardial Revascularization"[Mesh] or "Angioplasty, Balloon, Coronary"[Mesh] or "CABG"[tw]
#3 #1 OR #2
#4 "Surgical Procedures, Operative"[Mesh] or "Off-Pump Coronary Artery Bypass"[tw] or "Off-Pump Coronary Artery Bypass"[tw]
#5"Percutaneous Coronary Intervention"[Mesh] or "Robotic Surgical Procedures"[Mesh] or "Video-Assisted Surgery"[Mesh] or "Thoracoscopes"[Mesh] or "Thoracotomy"[Mesh]
#6 "Traditional thoracotomy"[tw] or "Conventional Surgery"[tw] or "Hybrid"[tw]
#7 #4 or #5 or #6
#8"random*"[tw] or "controlled"[tw] or "trial*"[tw] or "groups"[tw]
#9(("singl*"[tw] or "doubl*"[tw] or "tripl*"[tw]) and ("mask*"[tw] or "blind*"[tw]))
#10 #8 or #9
#11 #3 and #7 AND #10

Embase (Elsevier)

#1 ‘Coronary Disease’/exp
#2 ‘Coronary Artery Disease’/exp
#3 (“Left Main Diseases” or “Coronary Arteriosclerosis” or CABG): ti,ab
#4 #1 or #2 or #3
#5 ‘surgery’/exp
#6 “Operative Procedure”: ti,ab
#7 #5 or #6
#8‘Percutaneous Coronary Intervention’/exp
#9 “Percutaneous Coronary Revascularizations”: ti,ab
#10 #8 or #9
#11 ‘Robotic Surgical Procedures’/exp
#12 (“Robotic-Assisted Surgery” or “Robot Surgery”): ti,ab
#13 #11 or #12
#14“Video Assisted Surgery”: ti,ab
#15 ‘Video-Assisted Surgery’/exp
#16 #14 or #15
#17 ‘Thoracoscopes’/exp
#18 (“Pleuroscop*” or Thoracoscopy or “Endoscop*”): ti,ab

#19 #17 or #18
#20 ‘Thoracotomy’/exp
#21 (“Sternotom*” or “thoracotom*” or “hybrid”): ti,ab
#22 #20 or #21
#23 #7 or #10 or #13 or #16 or #19 or #22
#24 (“random*” or “control*” or “trial*” or placebo): ti,ab
#25 ((“singl*” or “doubl*” or “tripl*”) and (“mask*” or “blind*”)): ti,ab
#26 #23 or #24
#27 #4 AND #23 AND #26

Web of Sience

#1 “Coronary Artery Disease” or “Coronary Disease”
#2 “Left Main Disease*” or “Coronary Arteriosclerosis*”
#3 “Coronary Artery Bypass” or “Coronary Artery Bypass, Off Pump” or “Myocardial Revascularization”
#4 “Off-Pump Coronary Artery Bypass” or “Beating Heart Coronary Artery Bypass”
#5 #1 or #2 or #3 or #4
#6 “Surgical Procedures, Operative” or “Operative Surgical Procedure”
#7“Percutaneous Coronary Intervention” or “Percutaneous Coronary Revascularizations”
#8” Robot Surger*” or “Robotic-Assisted Surger*” or “Robotic Surgical Procedures”
#9” Video-Assisted Surger*” or “Video Assisted Surger*”
#10 “Thoracoscop*” or “Pleuroscope*” or “Endoscop*”
#11 “Thoracotom*” or “Thoracic Surgery” or “Sternotom*”
#12 #6 or #7 or #8 or #9 or #10 or #11
#13 #5 and #12

The Cochrane Library (Wiley Online Library)

#1 MeSH descriptor ‘Coronary Disease’ explode all trees
#2 (“Left Main Diseases” or “Coronary Arteriosclerosis”): ti,ab,kw
#3 MeSH descriptor ‘Coronary Artery Bypass’ explode all trees
#4 (“Off-Pump Coronary Artery Bypass” or “Beating Heart Coronary Artery Bypass”): ti,ab,kw
#5 #1 or #2 or #3 or #4
#6 MeSH descriptor ‘Operative Surgical Procedure’ explode all trees
#7 (“Operative Procedure*” or “Surgery, Ghost” or Surgery): ti,ab,kw
#9 MeSH descriptor’ Percutaneous Coronary Intervention’ explode all trees
#10 “Percutaneous Coronary Revascularizations”: ti,ab
#12 MeSH descriptor ‘Robotic Surgical Procedures’ explode all trees
#13 (“Robot Surger*” or “Robotic-Assisted Surgery*”): ti,ab
#14 MeSH descriptor ‘Video-Assisted Surgery’ explode all trees

#15 (“Video Assisted Surgery”: or Thoracoscopes): ti,ab,kw:
#16MeSH descriptor ‘Sternotomy’ explode all trees
#17 “Traditional thoracotomy” or “Median thoracotomy”: ti,ab,kw:
#18 #6 or #7 or #8 or #9 or #10 or #11 or #12 or #13 or #14 or #15 or #16 or #17
#19 #5 and #18 in Trials
