# Supplementary material for: Comparative efficacy of eight therapeutic methods in the treatment of left main coronary artery disease: a Bayesian network meta-analysis protocol
Source: BMJ Open. 2022 Sep 6;12(9):e058886. doi: 10.1136/bmjopen-2021-058886 (PMC9453992; doi:10.1136/bmjopen-2021-058886)
Supplement: Supplementary data [file bmjopen-2021-058886supp002.pdf]

PRISMA 2009 Flow Diagram

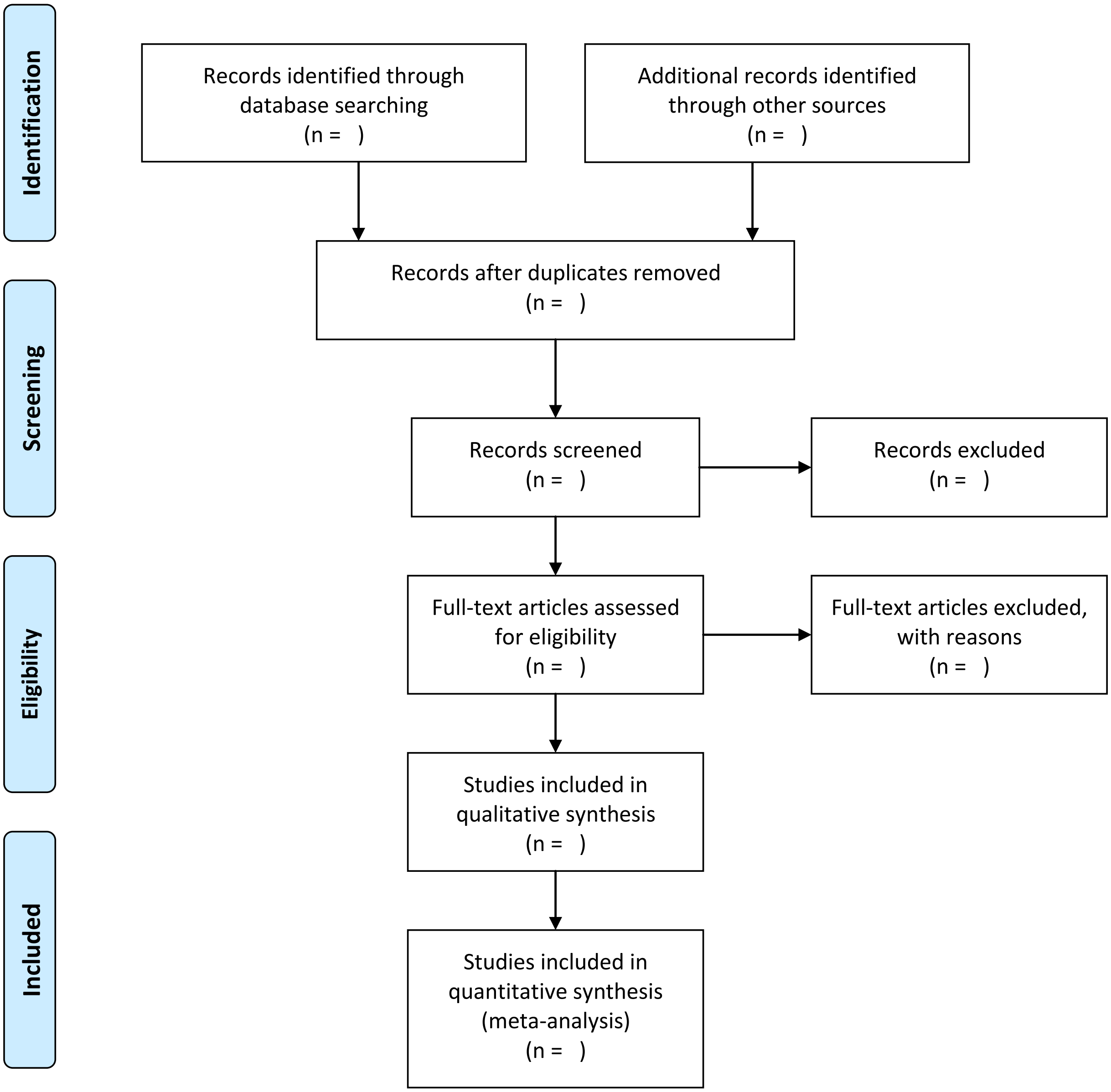

From: Moher D, Liberati A, Tetzlaff J, Altman DG, The PRISMA Group (2009). *P*referred *R*eporting *I*tems for *S*ystematic Reviews and *M*eta-*A*nalyses: The PRISMA Statement. PLoS Med 6(7): e1000097. doi:10.1371/journal.pmed1000097

For more information, visit www.prisma-statement.org.
