# Supplementary material for: Comparative efficacy of eight therapeutic methods in the treatment of left main coronary artery disease: a Bayesian network meta-analysis protocol
Source: BMJ Open. 2022 Sep 6;12(9):e058886. doi: 10.1136/bmjopen-2021-058886 (PMC9453992; doi:10.1136/bmjopen-2021-058886)
Supplement: Author's manuscript [file bmjopen-2021-058886.draft_revisions.pdf]

BMJ Open is committed to open peer review. As part of this commitment we make the peer review history of every article we publish publicly available.

When an article is published we post the peer reviewers' comments and the authors' responses online. We also post the versions of the paper that were used during peer review. These are the versions that the peer review comments apply to.

The versions of the paper that follow are the versions that were submitted during the peer review process. They are not the versions of record or the final published versions. They should not be cited or distributed as the published version of this manuscript.

BMJ Open is an open access journal and the full, final, typeset and author-corrected version of record of the manuscript is available on our site with no access controls, subscription charges or pay-per-view fees (http://bmjopen.bmj.com).

If you have any questions on BMJ Open's open peer review process please email info.bmjopen@bmj.com

BMJ Open

Comparative efficacy of 8 different surgical methods in the treatment of coronary heart disease: a Bayesian network meta-analysis protocol.

Journal:	*BMJ Open*
Manuscript ID	bmjopen-2021-058886
Article Type:	Protocol
Date Submitted by the Author:	31-Oct-2021

Complete List of Authors:	Hou, Biao; Inner Mongolia Medical University<br>Huang, Weimin; Inner Mongolia Medical University<br>Li, Qin; Inner Mongolia Medical University, Department of Cardiology, Baotou Central Hospital,Inner Mongolia Autonomous Region,China<br>Chen, Manlin; West China Hospital of Sichuan University<br>Wang, Liang; Baotou Central Hospital

Keywords:	Protocols & guidelines < HEALTH SERVICES ADMINISTRATION & MANAGEMENT, SURGERY, Coronary heart disease < CARDIOLOGY

SCHOLARONE™
Manuscripts

Comparative efficacy of 8 different surgical methods in the treatment of coronary heart disease: a Bayesian network meta-analysis protocol.

Biao Hou[1], Weimin Huang[1*], Qin Li[1], Manlin Chen[2], Liang Wang[3]

1: Inner Mongolia Medical University, Hohhot 010110, China.
2: West China Hospital of Sichuan University, Chengdu 610000, China.
3: Baotou Central Hospital, Baotou 014040, China.

BH(875125281@qq.com),QL(1797785034@qq.com),LW(1191051924@qq.com),MLC(6242020366@qq.com), WMH*(huang_manlin2021@163.com)
Mailing address: the sixth medical center of the General Hospital of the military region of the people's Liberation Army, No. 6 Fucheng Road, Haidian District, Beijing 100048, China

Abstract

Introduction As for coronary artery bypass grafting (CABG), although there are many direct comparative studies on different minimally invasive methods and traditional thoracotomy (off-pump / on-pump), there is still a lack of further ranking and summary of the efficacy of all surgical methods for left main branch lesions. Combined with the current controversial views, this study aims to introduce a planned network meta-analysis in detail, compare the long-term efficacy and safety of various surgical methods in the treatment of patients with coronary heart disease, and finally provide some reference basis for the best selection of clinical surgical schemes.

Method and analysis PubMed, Embase, Web of Science and The Cochrane Library databases will be collected from inception to June 2021 to compare the efficacy of different surgical methods in randomized controlled trials (RCTs) for coronary heart disease. Main outcome endpoints: Major adverse cardiovascular and cerebrovascular events (MACEs), including mortality, myocardial infarction, stroke and revascularization. Secondary outcome endpoints: (1) operation-related time, (2) blood transfusion rate, (3) complications including secondary thoracotomy, postoperative new atrial fibrillation, wound infection, (4) physiological score and psychological score, (5) time return to work, (6) total hospitalization costs. The methodological quality of included RCTs will be assessed according to the Cochrane bias risk table. The Bayesian network meta-analysis will be conducted by STATA 16.0.

Ethics and dissemination The essence of this study is to summarize and analyze the original data without the approval of the ethics committee. Our research does not involve ethical issues, and the results will be published in peer review journals.

PROSPERO registration number CRD42021274712

Strengths and limitations of this study:

1.This is the first Bayesian network analysis to comprehensively compare various coronary artery bypass grafting methods and the percutaneous coronary intervention (PCI).

2.The retrieval time is long, the scope is wide, and the quality of all included articles is strictly evaluated by two members with evidence-based medicine experience independently according to the manual.

3.There are mixed factors, such as different surgeon experience and population baseline characteristics, but the stability results of large samples of network analysis may conceal this effect.

For peer review only - http://bmjopen.bmj.com/site/about/guidelines.xhtml

INTRODUCTION

Left main coronary artery (LMCA) stenosis would involve large areas of myocardium and increase the risk of major adverse cardiac events[1]. LMCA treatment strategies include the CABG and the PCI. For more than 40 years, conventional extracorporeal circulation coronary artery bypass grafting (CECC) has been the gold standard for the treatment of LMCA diseases.[2] PCI is only used as a substitute for high-risk patients or not suitable for surgical patients[3]. There were some randomized controlled trials (EXCEL and NOBLE) on PCI and CABG[1 4 5], but the results showed some contradictory therapeutic outcomes.

In order to reduce the complications caused by extracorporeal circulation technology, off-pump coronary artery bypass grafting (OPCAB) has been carried out, and the relevant study[6] has shown that OPCAB can significantly reduce mortality and morbidity. However, some claim that OPCAB cannot provide the benefits of complete revascularization[7 8]. Others sought a compromise between the two surgeries, namely mini cardiopulmonary bypass coronary artery bypass (MECC), and there was a network meta-analysis[9] reported randomized controlled trials of this approach.

With the development of medical technology, other surgical methods for the treatment of coronary heart disease include : minimally invasive coronary artery bypass grafting under direct vision (MIDCAB) [10], robot-assisted coronary artery bypass grafting (RECAB)[11], total endoscopic coronary artery bypass grafting (TECAB)[12], and mixed coronary artery revascularization (HCR) [13], etc.

The different anatomical approaches of direct-viewing minimally invasive surgery may make surgeons feel stranger, and there are drawbacks that the assistants' vision is incomplete and unable to cooperate with them.[10] Similarly, RECAB and TECAB have higher technical requirements and long learning curve. If the operation is not smooth, the above methods are likely to be converted to the sternotomy approach.[11] For HCR, first of all, the sequence of PCI and CABG is currently controversial[14] ; secondly, the cost of hybrid technology is high, which is difficult for patients to accept and the promotion is limited[15].

Thus, under different circumstances, the best strategy for revascularization of left main lesions is still controversial. The purpose of this study is to summarize the above surgery methods for coronary heart disease, compare and rank them by using mesh meta-analysis, so as to provide some decision-making help for clinicians.

METHODS AND ANALYSIS

Literature Search

The protocol was formulated according to the 2015 checklist of the Preferred Reporting Items for Systematic Reviews and Meta-Analyses Protocols (PRISMA-P) [16 17]. The actual study will be implemented according to the PRISMA statement[18] and research guideline.[19]

Two authors (WMH and BH) will independently collect and screen RCTs on different surgical methods (including PCI) for the treatment of coronary heart disease from PubMed, Embase, Web of Science and The Cochrane Library databases. The search time limit is from the establishment of the database to June 2021.

The retrieval will be performed using a combination of grid words and free text words. Some English terms are "Coronary Disease, Left Main Disease, Coronary Artery Bypass, Myocardial Revascularization, CABG, Surgical Procedures, Percutaneous Coronary Intervention, Robotic Surgical Procedures, Video-Assisted Surgery, Thoracoscopes, Hybrid, Thoracotomy". The detailed search strategy is described in the ' online supplementary material appendix 1 '.

For peer review only - http://bmjopen.bmj.com/site/about/guidelines.xhtml

Eligibility criteria

Studies will be selected according to the PICO criteria: Patients (P), Intervention (I), Comparators(C), and Outcome(s) of interest (O).

Patients(P)： All the patients who participated in the study have undergone CABG or PCI for the first time. The only difference in population characteristics should be different surgical methods for the treatment of coronary heart disease. RCTs will be included in this meta-analysis.

Intervention(I)： The methods should include CECC, MECC, OPCAB, MIDCAB, RECAB, TECAB, HCR, and PCI.

Comparators(C)： The on-pump coronary artery bypass (ONCAB or CECC) operation method will be performed through median thoracotomy, which always used to the control to compare the main outcomes such as common postoperative complications, adverse cardiovascular and cerebrovascular events.

Outcomes(O): Primary Outcomes: Major adverse cardiovascular events (MACEs) including mortality, myocardial infarction, stroke, revascularization. Secondary Outcomes: (1) surgery-related time, (2) transfusion rate, (3) complications (4) physical score and psychological score, (5) time return to work, (6) total hospital costs. (Special definition: "complications" here refer to postoperative wound infection, pneumonia, liver and kidney dysfunction, new postoperative atrial fibrillation, etc. "physiological score and psychological score" are the scores determined by some literatures according to SF-12 and SF-36 quality of life questionnaire. The higher the score, the better the curative effect.)

Qualification criteria has been determined by two researchers (WMH and BH), and then discussed and agreed with other authors (QL, MLC and LW). As follows:

Inclusion criteria: (1) RCT trials; (2) All patients involved in the study were treated with CABG or PCI for the first time.

Exclusion criteria: (1) non-English literature; (2) patients with other major diseases that may affect the surgical efficacy (such as severe pulmonary hypertension); (3) unreasonable research design; (4) the full text or outcome indicators less than 3; (5) repeated publications by the same institution or author; (6) Continuity variables are not represented by mean ± standard (M±SD) deviation.

Selection process

Firstly, two authors (WMH and BH) will independently use the EndNote X9 software to classify and organize the searched literature according to surgical methods. Secondly, the excluded documents will be to place in a separate folder and marked to explain why they are excluded. The third step, by reading the titles and abstracts included in the literature, we would note the surgical grouping comparison (such as OPCAB vs MIDCAB) for future verification. The fourth step, by reading the full text, again exclude irrelevant literature, classify and mark. The fifth step is to judge by the third party (MLC or QL) if there is disagreement. We will strictly follow the above steps to ensure the high-quality and the comprehensiveness of the included literature.

Data extraction

The person responsible for screening (WMH and BH) will be asked to be familiar with the data in advance, and the data extraction table would be improved according to the situation, and the scoping studies will be conducted as recommended. [20]

For peer review only - http://bmjopen.bmj.com/site/about/guidelines.xhtml

The extracted data will include the publication years of the study, institutional background, random methods, baseline characteristics of patients (age, gender, body mass index (BMI), SYNTAX score, concomitant diseases, and number of blood revascularization), various outcome endpoints, missing visits, and statistical methods. In case of lack of data, we will contact the author by email.

Risk of bias in individual studies

Two reviewers (WMH and MLC) will be assessed. Any differences between reviewers will be resolved by discussing or requiring a third reviewer (BH) to assess. The included randomized controlled trials were independently assessed according to the Cochrane Handbook for Systematic Reviewers bias risk assessment criteria.[18] Each study will be graded by scores, as follows: A (low risk) : > 7 stars, B (medium risk) : 5-7 stars, C (high risk) : < 5 stars.[21]

Statistical analyses

In previous Meta-analysis publication[22] we used ADDIS software, it will be different next. We plan to use STATA16.0 software to draw a network diagram of the comparison of various interventions, and use Markov Chain Monte Carlo (MC-MC) method to simulate, the number of iterations is set to 50,000.[23] Interstudy will be evaluated by the Q statistic, where P<0.10 will be considered statistically significant and informative by I^2 statistic, where I^2≥50 % will indicate heterogeneity. We will perform subgroup and meta-analysis to assess differences.[24] In order to evaluate whether publication bias exists in the whole network, this study intends to use comparison-correction funnel plot[25]. The league table will be calculated for each main outcome endpoint, and the intervention measures were ranked according to the SUCRA value. The ranking results are reflected by the area under the cumulative ranking curve (SUCRA) [26].

The software to be used in this study are STATA 16.0 (Stata Corporation, College Station, TX 77845 USA) and Review Manager 5.4 (Oracle Corporation, The Cochrane Collaboration, 2020).

DISSCUSSION

As mentioned above, although the ONCAB or CECC has always been the gold standard for the treatment of LMCA diseases, with the rise of minimally invasive surgery, the discussion about the best strategy for revascularization of left main artery lesions is controversial in clinic.[2] Although numerous RCTs have compared CABG with PCI, no studies have been powered to detect a difference in mortality during the long follow-up among them. One study[27] has reported that no benefit for CABG over PCI was seen in patients with left main disease (CABG had a mortality benefit over PCI in patients with multivessel disease, and those with diabetes and higher coronary complexity.).

Although the current clinical guidelines have pointed that the SYNTAX score could help select the vascular reconstruction strategy for unprotected left main disease(ULMC)[28], one study of ten-year outcomes has shown that the discriminative capacity of SYNTAX score was relevant in the PCI group but not in the CABG group.[29]

Previous meta-analysis[9-13] showed that compared with traditional coronary artery bypass grafting, different surgical methods had certain advantages in different indicators. However, for the newly developed surgical treatment methods in recent years, such as robotic coronary artery bypass grafting[11], the number of randomized controlled trials is limited and lacks convincing, and there is no systematic and comprehensive comparison.

In order to ensure the quality of research, the authors will follow strict guidelines in the review process and their reports, such as PRISMA-P and PRISMA-ScR.[30] In order to avoid possible methodological defects, we

For peer review only - http://bmjopen.bmj.com/site/about/guidelines.xhtml

will use the latest guideline provided by The Joanna Briggs Institute (JBI) in 2020 when conducting the scope review.[31] Our proposed program was registered in a predefined manner to increase the transparency and reliability of the review results.[32]

Of course, our research also has limitations. For example, although there are extensive search strategies, we only include literature with English language. Others may worry that there are confounding factors, such as different surgeons experience, population baseline characteristics, etc., which make the results of the entire study different. However, as long as enough randomized controlled studies that meet the eligibility criteria are included, the stable results of the network analysis of large samples will mask this effect. In addition, when indirect comparison cannot be conducted in any case, we will conduct reliable direct comparison analysis results. If quantitative synthesis is not appropriate, narrative synthesis will be used.

In summary, the study planned by our team may be a comprehensive comparison of NMA in coronary artery bypass surgery. The analysis result will provide decision-making help for the best surgical choice of CABG or PCI.

Patient and public involvement

As the proposed systematic review will be conducted based on published studies, no patients and members of the public will be directly involved.

Amendments

Any amendments to this protocol will be documented.

Planned start and end date

The review is planned to start on 1 November 2021 and end on 1 June 2022.

Ethics and dissemination

The essence of this study is to summarize and analyze the original data without the approval of the ethics committee. Our research does not involve ethical issues, and the results will be published in peer review journals.

Contributors WMH: Concept research methodology, database search, article screening, data extraction, quality evaluation and drafting. BH: Database search, article screening and data extraction will be conducted. QL: Make the screening form, and judge the inconsistent opinions. MLC: Literature quality evaluation and statistical analysis. LW: Participate in the outcome discussion. All authors will read and approve the final manuscript.

Funding This work was supported by Baotou Science and Technology Plan (2017Y2012).

Competing interests None declared.

Patient consent for publication Not applicable.

Provenance and peer review Not commissioned; external peer review

Open access This is an open access article distributed in accordance with the Creative Commons Attribution non-Commercial (CC BY- NC 4.0) license, which permits others to distribute, remix, adapt, build upon this work non- commercially, and license their derivative works on different terms, provided the original work is properly cited, appropriate credit is given, any changes made indicated, and the use is non- commercial. See: http://creativecommons. org/ licenses/ by-nc/ 4. 0/.

ORCID iDs

Weimin Huang http://orcid.org/0000-0003-2556-6214

For peer review only - http://bmjopen.bmj.com/site/about/guidelines.xhtml

REFERENCES

1. Holm NR, Makikallio T, Lindsay MM, et al. Percutaneous coronary angioplasty versus coronary artery bypass grafting in the treatment of unprotected left main stenosis: updated 5-year outcomes from the randomised, non-inferiority NOBLE trial. *Lancet* 2020;395(10219):191-99. doi: 10.1016/S0140-6736(19)32972-1 [published Online First: 2019/12/28]

2. Alasnag M, Yaqoub L, Saati A, et al. Left Main Coronary Artery Interventions. *Interv Cardiol* 2019;14(3):124-30. doi: 10.15420/icr.2019.10.R2 [published Online First: 2019/12/25]

3. Daemen J, Boersma E, Flather M, et al. Long-term safety and efficacy of percutaneous coronary intervention with stenting and coronary artery bypass surgery for multivessel coronary artery disease: a meta-analysis with 5-year patient-level data from the ARTS, ERACI-II, MASS-II, and SoS trials. *Circulation* 2008;118(11):1146-54. doi: 10.1161/CIRCULATIONAHA.107.752147 [published Online First: 2008/08/30]

4. Doucet S, Jolicoeur EM, Serruys PW, et al. Outcomes of left main revascularization in patients with acute coronary syndromes and stable ischemic heart disease: Analysis from the EXCEL trial. *Am Heart J* 2019;214:9-17. doi: 10.1016/j.ahj.2019.04.016 [published Online First: 2019/06/01]

5. Stone GW, Kappetein AP, Sabik JF, et al. Five-Year Outcomes after PCI or CABG for Left Main Coronary Disease. *N Engl J Med* 2019;381(19):1820-30. doi: 10.1056/NEJMoa1909406 [published Online First: 2019/09/29]

6. Marui A, Okabayashi H, Komiya T, et al. Benefits of off-pump coronary artery bypass grafting in high-risk patients. *Circulation* 2012;126(11 Suppl 1):S151-7. doi: 10.1161/CIRCULATIONAHA.111.083873 [published Online First: 2012/11/06]

7. Zubarevich A, Kadyraliev B, Arutyunyan V, et al. On-pump versus off-pump coronary artery bypass surgery for multi-vessel coronary revascularization. *J Thorac Dis* 2020;12(10):5639-46. doi: 10.21037/jtd-20-1284 [published Online First: 2020/11/20]

8. Shroyer AL, Grover FL, Hattler B, et al. On-pump versus off-pump coronary-artery bypass surgery. *N Engl J Med* 2009;361(19):1827-37. doi: 10.1056/NEJMoa0902905 [published Online First: 2009/11/06]

9. Kowalewski M, Pawliszak W, Raffa GM, et al. Safety and efficacy of miniaturized extracorporeal circulation when compared with off-pump and conventional coronary artery bypass grafting: evidence synthesis from a comprehensive Bayesian-framework network meta-analysis of 134 randomized controlled trials involving 22 778 patients. *European journal of cardio-thoracic surgery : official journal of the European Association for Cardio-thoracic Surgery* 2016;49(5):1428-40. doi: 10.1093/ejcts/ezv387 [published Online First: 2015/11/06]

10. Indja B, Woldendorp K, Black D, et al. Minimally invasive surgical approaches to left main and left anterior descending coronary artery revascularization are superior compared to first- and second-generation drug-eluting stents: a network meta-analysis. *European journal of cardio-thoracic surgery : official journal of the European Association for Cardio-thoracic Surgery* 2020;57(1):18-27. doi: 10.1093/ejcts/ezz184 [published Online First: 2019/06/21]

11. Hammal F, Nagase F, Menon D, et al. Robot-assisted coronary artery bypass surgery: a systematic review and meta-analysis of comparative studies. *Canadian journal of surgery Journal canadien de chirurgie* 2020;63(6):E491-E508. [published Online First: 2020/11/07]

12. Leonard JR, Rahouma M, Abouarab AA, et al. Totally endoscopic coronary artery bypass surgery: A meta-analysis of the current evidence. *Int J Cardiol* 2018;261:42-46. doi: 10.1016/j.ijcard.2017.12.071 [published Online First: 2018/04/17]

13. Reynolds AC, King N. Hybrid coronary revascularization versus conventional coronary artery bypass grafting: Systematic review and meta-analysis. *Medicine (Baltimore)* 2018;97(33):e11941. doi: 10.1097/MD.0000000000011941 [published Online First: 2018/08/17]

14. Kayatta MO, Halkos ME. Reviewing hybrid coronary revascularization: challenges, controversies and opportunities. *Expert Rev Cardiovasc Ther* 2016;14(7):821-30. doi: 10.1080/14779072.2016.1174576 [published Online First: 2016/04/05]

15. Zhou S, Fang Z, Xiong H, et al. Effect of one-stop hybrid coronary revascularization on postoperative renal function and bleeding: a comparison study with off-pump coronary artery bypass grafting surgery. *The Journal of thoracic and cardiovascular surgery* 2014;147(5):1511-16 e1. doi: 10.1016/j.jtcvs.2013.05.026 [published Online First: 2013/07/25]

16. Shamseer L, Moher D, Clarke M, et al. Preferred reporting items for systematic review and meta-analysis protocols (PRISMA-P) 2015: elaboration and explanation. *BMJ* 2015;350:g7647. doi: 10.1136/bmj.g7647 [published Online First: 2015/01/04]

For peer review only - http://bmjopen.bmj.com/site/about/guidelines.xhtml

17. Moher D, Shamseer L, Clarke M, et al. Preferred reporting items for systematic review and meta-analysis protocols (PRISMA-P) 2015 statement. *Syst Rev* 2015;4:1. doi: 10.1186/2046-4053-4-1 [published Online First: 2015/01/03]

18. Higgins JP, Altman DG, Gotzsche PC, et al. The Cochrane Collaboration's tool for assessing risk of bias in randomised trials. *BMJ* 2011;343:d5928. doi: 10.1136/bmj.d5928 [published Online First: 2011/10/20]

19. Page MJ, McKenzie JE, Bossuyt PM, et al. The PRISMA 2020 statement: an updated guideline for reporting systematic reviews. *BMJ* 2021;372:n71. doi: 10.1136/bmj.n71 [published Online First: 2021/03/31]

20. Levac D, Colquhoun H, O'Brien KK. Scoping studies: advancing the methodology. *Implement Sci* 2010;5:69. doi: 10.1186/1748-5908-5-69 [published Online First: 2010/09/22]

21. Guyatt G, Oxman AD, Akl EA, et al. GRADE guidelines: 1. Introduction-GRADE evidence profiles and summary of findings tables. *J Clin Epidemiol* 2011;64(4):383-94. doi: 10.1016/j.jclinepi.2010.04.026 [published Online First: 2011/01/05]

22. Huang W, Hou B, Li Q, et al. Comparative efficacy of five surgical methods in the treatment of mitral regurgitation: A systematic review and network meta-analysis. *J Card Surg* 2021 doi: 10.1111/jocs.16085 [published Online First: 2021/10/19]

23. Wang J, Zhu X, Sun Y, et al. Efficacy and safety of traditional Chinese medicine combined with routine western medicine for the asymptomatic novel coronavirus disease (COVID-19): A Bayesian network meta-analysis protocol. *Medicine (Baltimore)* 2020;99(35):e21927. doi: 10.1097/MD.0000000000021927 [published Online First: 2020/09/03]

24. Brignardello-Petersen R, Izcovich A, Rochwerg B, et al. GRADE approach to drawing conclusions from a network meta-analysis using a partially contextualised framework. *BMJ* 2020;371:m3907. doi: 10.1136/bmj.m3907 [published Online First: 2020/11/12]

25. Shim S, Yoon BH, Shin IS, et al. Network meta-analysis: application and practice using Stata. *Epidemiol Health* 2017;39:e2017047. doi: 10.4178/epih.e2017047 [published Online First: 2017/11/03]

26. Kuo FC, Hsu CW, Tan TL, et al. Effectiveness of Different Wound Dressings in the Reduction of Blisters and Periprosthetic Joint Infection After Total Joint Arthroplasty: A Systematic Review and Network Meta-Analysis. *J Arthroplasty* 2021;36(7):2612-29. doi: 10.1016/j.arth.2021.02.047 [published Online First: 2021/03/13]

27. Head SJ, Milojevic M, Daemen J, et al. Mortality after coronary artery bypass grafting versus percutaneous coronary intervention with stenting for coronary artery disease: a pooled analysis of individual patient data. *Lancet* 2018;391(10124):939-48. doi: 10.1016/S0140-6736(18)30423-9 [published Online First: 2018/02/27]

28. Freemantle N, Pagano D. Concerns with the new SYNTAX score. *Lancet* 2021;397(10276):795. doi: 10.1016/S0140-6736(21)00223-3 [published Online First: 2021/03/01]

29. Yoon YH, Ahn JM, Kang DY, et al. Impact of SYNTAX Score on 10-Year Outcomes After Revascularization for Left Main Coronary Artery Disease. *JACC Cardiovasc Interv* 2020;13(3):361-71. doi: 10.1016/j.jcin.2019.10.020 [published Online First: 2020/02/08]

30. Tricco AC, Lillie E, Zarin W, et al. PRISMA Extension for Scoping Reviews (PRISMA-ScR): Checklist and Explanation. *Ann Intern Med* 2018;169(7):467-73. doi: 10.7326/M18-0850 [published Online First: 2018/09/05]

31. Peters MDJ, Marnie C, Tricco AC, et al. Updated methodological guidance for the conduct of scoping reviews. *JBI Evid Synth* 2020;18(10):2119-26. doi: 10.11124/JBIES-20-00167 [published Online First: 2020/10/11]

32. Page MJ, Shamseer L, Tricco AC. Registration of systematic reviews in PROSPERO: 30,000 records and counting. *Syst Rev* 2018;7(1):32. doi: 10.1186/s13643-018-0699-4 [published Online First: 2018/02/22]

For peer review only - http://bmjopen.bmj.com/site/about/guidelines.xhtml

Appendix: Search Strategies

PubMed

#1"Coronary Disease"[Mesh] or "Left Main Disease*"[tw] or "Coronary Arteriosclerosis*"[tw]
#2"Coronary Artery Bypass"[Mesh] or "Myocardial Revascularization"[Mesh] or "Angioplasty, Balloon, Coronary"[Mesh] or "CABG"[tw]
#3 #1 OR #2
#4 "Surgical Procedures, Operative"[Mesh] or "Off-Pump Coronary Artery Bypass"[tw] or "Off-Pump Coronary Artery Bypass"[tw]
#5"Percutaneous Coronary Intervention"[Mesh] or "Robotic Surgical Procedures"[Mesh] or "Video-Assisted Surgery"[Mesh] or "Thoracoscopes"[Mesh] or "Thoracotomy"[Mesh]
#6 "Traditional thoracotomy"[tw] or "Conventional Surgery"[tw] or "Hybrid"[tw]
#7 #4 or #5 or #6
#8"random*"[tw] or "controlled"[tw] or "trial*"[tw] or "groups"[tw]
#9(("singl*"[tw] or "doubl*"[tw] or "tripl*"[tw]) and ("mask*"[tw] or "blind*"[tw]))
#10 #8 or #9
#11 #3 and #7 AND #10

Embase (Elsevier)

#1 'Coronary Disease'/exp
#2 'Coronary Artery Disease'/exp
#3 ("Left Main Diseases" or "Coronary Arteriosclerosis" or CABG): ti,ab
#4 #1 or #2 or #3
#5 'surgery'/exp
#6 "Operative Procedure": ti,ab
#7 #5 or #6
#8'Percutaneous Coronary Intervention'/exp
#9 "Percutaneous Coronary Revascularizations": ti,ab
#10 #8 or #9
#11 'Robotic Surgical Procedures'/exp
#12 ("Robotic-Assisted Surgery" or "Robot Surgery"): ti,ab
#13 #11 or #12
#14"Video Assisted Surgery": ti,ab
#15 'Video-Assisted Surgery'/exp
#16 #14 or #15
#17 'Thoracoscopes'/exp
#18 ("Pleuroscop*" or Thoracoscopy or "Endoscop*"): ti,ab

For peer review only

#19 #17 or #18
#20 ‘Thoracotomy’/exp
#21 (“Sternotom*” or “thoracotom*” or “hybrid”): ti,ab
#22 #20 or #21
#23 #7 or #10 or #13 or #16 or #19 or #22
#24 (“random*” or “control*” or “trial*” or placebo): ti,ab
#25 ((“singl*” or “doubl*” or “tripl*”) and (“mask*” or “blind*”)): ti,ab
#26 #23 or #24
#27 #4 AND #23 AND #26

Web of Sience

#1 “Coronary Artery Disease” or “Coronary Disease”
#2 “Left Main Disease*” or “Coronary Arteriosclerosis*”
#3 “Coronary Artery Bypass” or “Coronary Artery Bypass, Off Pump” or “Myocardial Revascularization”
#4 “Off-Pump Coronary Artery Bypass” or “Beating Heart Coronary Artery Bypass”
#5 #1 or #2 or #3 or #4
#6 “Surgical Procedures, Operative” or “Operative Surgical Procedure”
#7“Percutaneous Coronary Intervention” or “Percutaneous Coronary Revascularizations”
#8” Robot Surger*” or “Robotic-Assisted Surger*” or “Robotic Surgical Procedures”
#9” Video-Assisted Surger*” or “Video Assisted Surger*”
#10 “Thoracoscop*” or “Pleuroscope*” or “Endoscop*”
#11 “Thoracotom*” or “Thoracic Surgery” or “Sternotom*”
#12 #6 or #7 or #8 or #9 or #10 or #11
#13 #5 and #12

The Cochrane Library (Wiley Online Library)

#1 MeSH descriptor ‘Coronary Disease’ explode all trees
#2 (“Left Main Diseases” or “Coronary Arteriosclerosis”): ti,ab,kw
#3 MeSH descriptor ‘Coronary Artery Bypass’ explode all trees
#4 (“Off-Pump Coronary Artery Bypass” or “Beating Heart Coronary Artery Bypass”): ti,ab,kw
#5 #1 or #2 or #3 or #4
#6 MeSH descriptor ‘Operative Surgical Procedure’ explode all trees
#7 (“Operative Procedure*” or “Surgery, Ghost” or Surgery): ti,ab,kw
#9 MeSH descriptor’ Percutaneous Coronary Intervention’ explode all trees
#10 “Percutaneous Coronary Revascularizations”: ti,ab
#12 MeSH descriptor ‘Robotic Surgical Procedures’ explode all trees
#13 (“Robot Surger*” or “Robotic-Assisted Surgery*”): ti,ab
#14 MeSH descriptor ‘Video-Assisted Surgery’ explode all trees

For peer review only

#15 (“Video Assisted Surgery”: or Thoracoscopes): ti,ab,kw:
#16MeSH descriptor ‘Sternotomy’ explode all trees
#17 “Traditional thoracotomy” or “Median thoracotomy”: ti,ab,kw:
#18 #6 or #7 or #8 or #9 or #10 or #11 or #12 or #13 or #14 or #15 or #16 or #17
#19 #5 and #18 in Trials

For peer review only

PRISMA-P (Preferred Reporting Items for Systematic review and Meta-Analysis Protocols) 2015 checklist: recommended items to address in a systematic review protocol*

Section and topic	Item No	Checklist item
**ADMINISTRATIVE INFORMATION**		
Title:		
Identification	1a	Comparative efficacy of 8 different surgical methods in the treatment of coronary heart disease: a Bayesian network meta-analysis protocol (Page 1 line 1-2)
Update	1b	None
Registration	2	PROSPERO registration number CRD42021274712(Page 1 line 35)
Authors:		
Contact	3a	All names, institutional affiliations, e-mail address of all protocol authors are provided as well as physical mailing address of corresponding author (Page 1 line 4-13)
Contributions	3b	The contributions of protocol authors are listed and the guarantor of the review is identified (Page 5 line 32-36)
Amendments	4	Amendments are not expected but all deviations will be documented and discussed (Page 5 line 20-21)
Support:		
Sources	5a	This work was supported by Baotou Science and Technology Planning Project (2017y2012)
Sponsor	5b	Individual: Huang Weimin, the researcher of the team. (Page 1, 5)
Role of sponsor or funder	5c	The funders had no role in the study design and will not have any role during its execution, analysis, interpretation of the data, decision to publish, or preparation of the manuscript. (Page 5)
**INTRODUCTION**		
Rationale	6	The rationale for the review is described in contrast to what is already known and the gaps in literature (Page 2 line 1-31)
Objectives	7	We provided our explicit objectives (Page 1) and the participants, interventions, comparators, and outcomes (PICO) on (Page 3 line 2-22)
**METHODS**		
Eligibility criteria	8	We explicitly described our inclusion and exclusion criteria (Page 3). (such as PICO, study design, setting, time frame) and report characteristics (such as years considered, language, publication status) to be used as criteria for eligibility for the review (Page 3 line 24-32)
Information sources	9	We described our search strategy, databases that will be used and data sources (Page 2)
Search strategy	10	The search strategy to be used for 4 electronic databases, including planned limits, such that it could be repeated. (Page 2)
Study records:		
Data management	11a	Endnote software was used to classify and arrange the searched literature according to the surgical method (Page 3 line

For peer review only

		35-41)
Selection process	11b	We clearly state the process that will be used for selecting studies (Page 3)
Data collection process	11c	We described the plan of extracting data from reports (Page 3-4)
Data items	12	We listed and defined all variables for which data will be sought (Page 4 line 1-4)
Outcomes and prioritization	13	We listed and defined all outcomes for which data will be sought, including prioritization of main and additional outcomes, with rationale (Page 1, 3)
Risk of bias in individual studies	14	We described anticipated methods for assessing risk of bias of individual studies, including whether this will be done at the outcome or study level. We may use the Review Manager software. (Page 4 line 6-11)
Data synthesis	15a	We described criteria under which study data will be quantitatively synthesized (Page 3 line 1-4)
	15b	We described our plan to assess heterogeneity (Page 4 line 17-19)
	15c	We describe our additional analyses (including sensitivity, subgroup analyses, and meta-regression) (Page 4-5)
	15d	If quantitative synthesis is not appropriate, narrative synthesis will be used. (Page 5 line 11-12)
Meta-bias(es)	16	We described the meta-bias (Page 4 line 20)
Confidence in cumulative evidence	17	We will use a quality score as described. (Page 4 line 8-10)

*** It is strongly recommended that this checklist be read in conjunction with the PRISMA-P Explanation and Elaboration (cite when available) for important clarification on the items. Amendments to a review protocol should be tracked and dated. The copyright for PRISMA-P (including checklist) is held by the PRISMA-P Group and is distributed under a Creative Commons Attribution Licence 4.0.**

From: Shamseer L, Moher D, Clarke M, Ghersi D, Liberati A, Petticrew M, Shekelle P, Stewart L, PRISMA-P Group. Preferred reporting items for systematic review and meta-analysis protocols (PRISMA-P) 2015: elaboration and explanation. BMJ. 2015 Jan 2;349(jan02 1):g7647.

For peer review only

PRISMA 2009 Flow Diagram

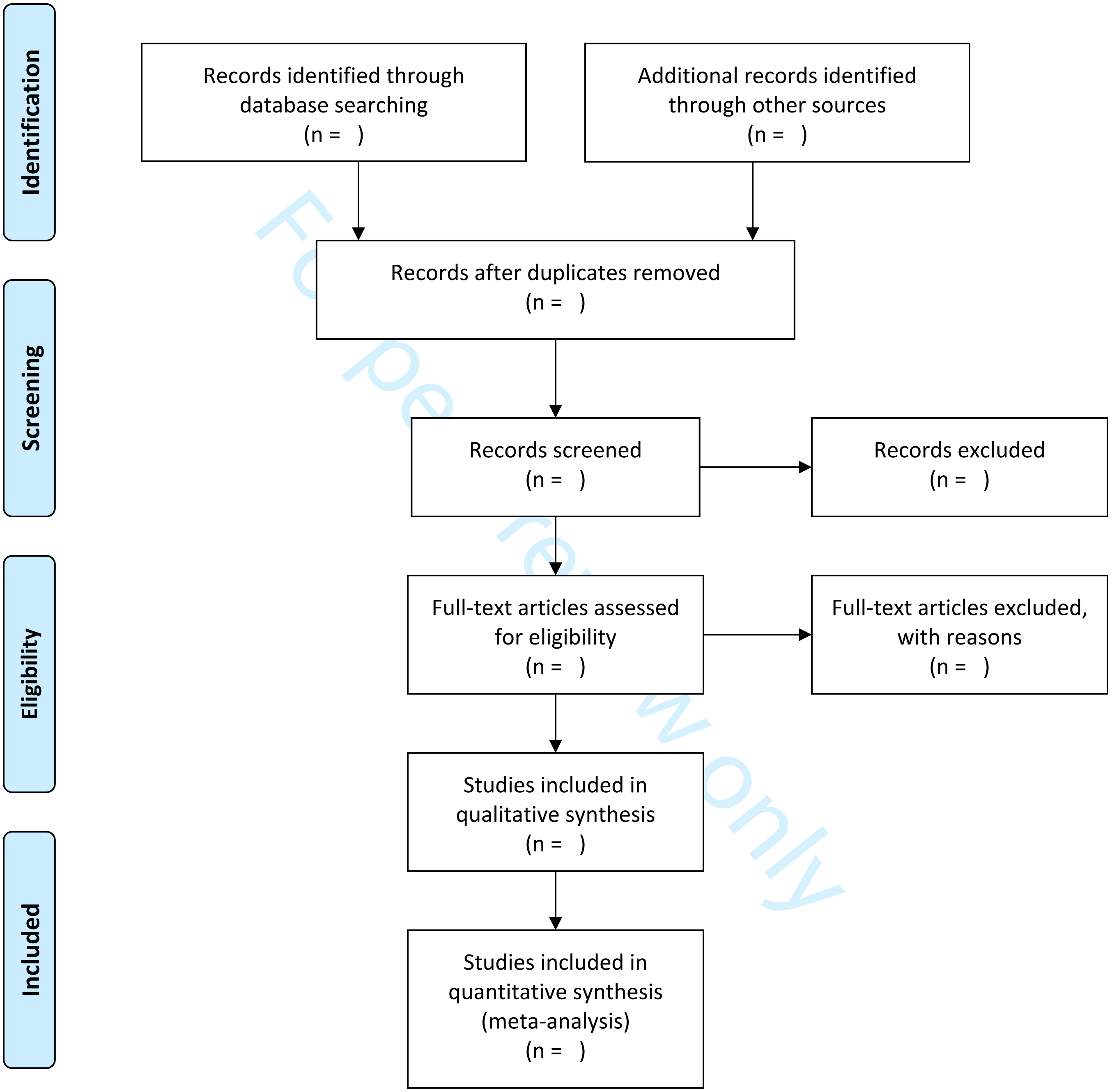

From: Moher D, Liberati A, Tetzlaff J, Altman DG, The PRISMA Group (2009). *P*referred *R*eporting *I*tems for *S*ystematic Reviews and *M*eta-*A*nalyses: The PRISMA Statement. PLoS Med 6(7): e1000097. doi:10.1371/journal.pmed1000097

For more information, visit www.prisma-statement.org.

For peer review only - http://bmjopen.bmj.com/site/about/guidelines.xhtml

BMJ 2016;354:i4086 doi: 10.1136/bmj.i4086 (Published 21 July 2016)

CORRECTIONS

Preferred reporting items for systematic review and meta-analysis protocols (PRISMA-P) 2015: elaboration and explanation

This Research Methods and Reporting paper (*BMJ* 2015;350:g7647, doi:10.1136/bmj.g7647) should have the volume number 350 (not 349).

For peer review only

For personal use only: See rights and reprints http://www.bmj.com/permissions Subscribe: http://www.bmj.com/subscribe

Comparative efficacy of 8 therapeutic methods in the treatment of left main coronary artery disease: a Bayesian network meta-analysis protocol.

Journal:	*BMJ Open*
Manuscript ID	bmjopen-2021-058886.R1
Article Type:	Protocol
Date Submitted by the Author:	30-Jun-2022
Complete List of Authors:	Hou, Biao; Inner Mongolia Medical University<br>Chen, Manlin; Bazhong Central Hospital<br>Li, Qin; Inner Mongolia Medical University, Department of Cardiology, Baotou Central Hospital,Inner Mongolia Autonomous Region,China<br>Huang, Weimin; Inner Mongolia Medical University<br>Wang, Liang; Inner Mongolia Medical University; Inner Mongolia Baotou City Central Hospital
**Primary Subject Heading**:	Cardiovascular medicine
Secondary Subject Heading:	Surgery
Keywords:	Protocols & guidelines < HEALTH SERVICES ADMINISTRATION & MANAGEMENT, SURGERY, Coronary heart disease < CARDIOLOGY

Comparative efficacy of 8 therapeutic methods in the treatment of left main coronary artery disease: a Bayesian network meta-analysis protocol.

Biao Hou[1], Manlin Chen[2], Qin Li[1], Weimin Huang[1,3*], Liang Wang[1,3]

1: Inner Mongolia Medical University, Hohhot 010110, China.
2: Bazhong Central Hospital, Bazhong 636600, China.
3: Baotou Central Hospital, Baotou 014040, China.

BH(dr_biao@163.com),QL(1797785034@qq.com),MLC(624202366@qq.com),WMH*(huang_manlin2021@163.com) ,LW(wangliangtong@qq.com)
Mailing address: No. 61, Huancheng Road, Donghe District, Baotou 014040, China.
Word count – 2311words (excluding title page, references, figures)

Abstract

Introduction As for coronary artery bypass grafting (CABG), although there are many direct comparative studies on different minimally invasive methods and traditional thoracotomy (off-pump / on-pump), there is still a lack of further ranking and summary of the efficacy of all surgical methods for left main coronary artery (LMCA) lesions. Combined with the current controversial views, this study aims to introduce a planned network meta-analysis (NMA) in detail, with a view to comparing the long-term efficacy and safety of multiple therapeutic methods in the treatment of patients with LMCA disease, and finally providing some reference bases for the best selection of clinical schemes.

Method and analysis PubMed, Embase, Web of Science and The Cochrane Library databases will be collected from inception to November 2021 to compare the efficacy of different surgical methods in randomized controlled trials (RCTs) for left main coronary artery disease. Main outcome endpoints: Major adverse cardiovascular events (MACEs), including mortality, myocardial infarction, stroke and revascularization. Secondary outcome endpoints: (1) operation-related time, (2) the amount of blood transfusion, (3) complications including secondary thoracotomy, postoperative new atrial fibrillation, wound infection, (4) physiological score and psychological score, (5) time return to work, (6) total hospitalization costs. The methodological quality of included RCTs will be assessed according to the Cochrane bias risk table. The Bayesian network meta-analysis will be conducted by STATA 16.0.

Ethics and dissemination The essence of this study is to summarize and analyze the original data without the approval of the ethics committee. Our research does not involve ethical issues, and the results will be published in peer review journals.

PROSPERO registration number CRD42021274712

Keywords: Protocols & guidelines; SURGERY; Coronary heart disease

Strengths and limitations of this study:

1. This is the one Bayesian network analysis to comprehensively compare various therapeutic methods of left main coronary artery (LMCA) disease.
2. The retrieval time is long, the scope is wide, and the quality of all included articles will be strictly evaluated by two members with evidence-based medicine experience independently according to the manual.
3. There may be mixed factors, such as different surgeon experience and population baseline characteristics, but comprehensive analysis methods such as subgroup analysis and the stability results of large samples may conceal this effect.

For peer review only - http://bmjopen.bmj.com/site/about/guidelines.xhtml

INTRODUCTION

Left main coronary artery (LMCA) stenosis would involve large areas of myocardium and increase the risk of major adverse cardiac events[1]. LMCA treatment strategies include the CABG and the PCI. For more than 40 years, conventional extracorporeal circulation coronary artery bypass grafting (CECC) has been the gold standard for the treatment of LMCA diseases.[2] PCI was only used as a substitute for high-risk patients or not suitable for surgical patients[3]. There were some randomized controlled trials on PCI and CABG[1 4 5], but the results showed some contradictory therapeutic outcomes.

In order to reduce the complications caused by extracorporeal circulation technology, off-pump coronary artery bypass grafting (OPCAB) has been carried out, and the relevant study[6] has shown that OPCAB can significantly reduce mortality and morbidity. However, some claim that OPCAB cannot provide the benefits of complete revascularization[7 8]. Others sought a compromise between the two surgeries, namely mini cardiopulmonary bypass coronary artery bypass (MECC), and there was a network meta-analysis[9] reported randomized controlled trials of this approach.

With the development of medical technology, other surgical methods for the treatment of coronary heart disease include : minimally invasive coronary artery bypass grafting under direct vision (MIDCAB) [10], robot-assisted coronary artery bypass grafting (RECAB)[11], total endoscopic coronary artery bypass grafting (TECAB)[12], and hybrid coronary artery revascularization (HCR) [13], etc.

The different anatomical approaches of direct-viewing minimally invasive surgery may make surgeons feel stranger, and there are drawbacks that the assistants' vision is incomplete and unable to cooperate with them.[10] Similarly, RECAB and TECAB technologies both need higher technical threshold requirements and longer learning curve. If the key process of operation is not smooth, the above methods are likely to be converted to the sternotomy approach.[11] For HCR, first of all, the sequence of PCI and CABG is currently controversial[14] ; secondly, the cost of hybrid technology is high, which is difficult for patients to accept and the promotion is limited[15].

Thus, under different circumstances, the best strategy for revascularization of left main lesions is still controversial. The purpose of this study is to summarize the above surgery methods for coronary heart disease, compare and rank them by using mesh meta-analysis, so as to provide some decision-making help for clinicians.

METHODS AND ANALYSIS

Literature Search

The protocol was formulated according to the 2015 checklist of the Preferred Reporting Items for Systematic Reviews and Meta-Analyses Protocols (PRISMA-P) [16 17]. The actual study will be implemented according to the PRISMA statement[18] and research guideline.[19]

Two authors (WMH and BH) will independently collect and screen RCTs on different surgical methods (including PCI) for the treatment of coronary heart disease from PubMed, Embase, Web of Science and The Cochrane Library databases. The search time limit is from the establishment of the database to January 2023.

The retrieval will be performed using a combination of grid words and free text words. Some English terms are "Coronary Disease, Left Main Disease, Coronary Artery Bypass, Myocardial Revascularization, CABG, Surgical Procedures, Percutaneous Coronary Intervention, Robotic Surgical Procedures, Video-Assisted

For peer review only - http://bmjopen.bmj.com/site/about/guidelines.xhtml

Surgery, Thoracoscopes, Hybrid, Thoracotomy". The detailed search strategy is described in the ' online supplementary material appendix 1 '.

Eligibility criteria

Studies will be selected according to the PICO criteria: Patients (P), Intervention (I), Comparators(C), and Outcome(s) of interest (O).

Patients(P)： All patients who were included in the study have undergone CABG or PCI for the first time. The only difference in population characteristics should be different treatment methods of coronary heart disease. RCTs will be included in this meta-analysis.

Intervention(I)： The methods should include CECC, MECC, OPCAB, MIDCAB, RECAB, TECAB, HCR, and PCI.

Comparators(C)： The on-pump coronary artery bypass (ONCAB or CECC) operation method will be performed through median thoracotomy, which always used to the control to compare the main outcomes such as common postoperative complications, adverse cardiovascular and cerebrovascular events.

Outcomes(O): Primary Outcomes: MACE endpoints should include the numbers of mortality, myocardial infarction, stroke, revascularization during the follow-up time which is at least 1 year. The occurrence of adverse events can be counted respectively during their hospitalization, 6 months after operation, 1 year or more after operation. Secondary Outcomes: (1) surgery-related time, (2) the amount of blood transfusion, (3) complications (4) physical score and psychological score, (5) time return to work, (6) total hospital costs. (Special definition: "blood transfusion": it should include the amount of blood transfusion during the operation and during the stay in the Cardiac Surgical Intensive Care Unit (CSICU). It often refers to the cumulative amount of blood transfusion during the hospitalization. "complications" here refer to postoperative wound infection, pneumonia, liver and kidney dysfunction, new postoperative atrial fibrillation, etc. "physiological score and psychological score" are the scores determined by some literatures according to SF-12 and SF-36 quality of life questionnaire. The higher the score, the better the curative effect.)

Qualification criteria has been determined by two researchers (WMH and BH), and then discussed and agreed with other authors (QL, MLC and LW). As follows:

Inclusion criteria: (1) RCT trials; (2) All patients involved in the study were treated with CABG or PCI for the first time.

Exclusion criteria: (1) non-English literature; (2) patients with other major diseases that may affect the surgical efficacy (such as severe pulmonary hypertension); (3) unreasonable research design; (4) the full text or outcome indicators less than 3; (5) repeated publications by the same institution or author; (6) Continuity variables are not represented by mean ± standard (M±SD) deviation.

Selection process

Firstly, two authors (WMH and BH) will independently use the EndNote X9 software to classify and organize the searched literature according to surgical methods. Secondly, the excluded documents will be to place in a separate folder and marked to explain why they are excluded. The third step, by reading the titles and abstracts included in the literature, we would note the surgical grouping comparison (such as OPCAB vs MIDCAB) for future verification. The fourth step, by reading the full text, again exclude irrelevant literature, classify and

For peer review only - http://bmjopen.bmj.com/site/about/guidelines.xhtml

mark. The fifth step is to judge by the third party (MLC or QL) if there is disagreement. We will strictly follow the above steps to ensure the high-quality and the comprehensiveness of the included literature.

Data extraction

The person responsible for screening (WMH and BH) will be asked to be familiar with the data in advance, and the data extraction table would be improved according to the situation, and the scoping studies will be conducted as recommended. [20]

The extracted data will include the publication years of the study, institutional background, random methods, baseline characteristics of patients (age, gender, the body mass index (BMI), the SYNTAX score, concomitant diseases, and the number of revascularized vessels), various outcome endpoints, missing visits, and statistical methods. In addition, we will collect data on the type of surgery (elective, urgent or emergency), surgical indications (acute vs chronic coronary syndrome), and the medical therapy patients (antiplatelet therapy) during the perioperative period, as appropriate. In case of lack of data, we will contact the author by email.

Risk of bias in individual studies

Two reviewers (WMH and MLC) will be assessed. Any differences between reviewers will be resolved by discussing or requiring a third reviewer (BH) to assess. The included randomized controlled trials were independently assessed according to the Cochrane Handbook for Systematic Reviewers bias risk assessment criteria.[18] Each study will be graded by scores, as follows: A (low risk) : > 7 stars, B (medium risk) : 5-7 stars, C (high risk) : < 5 stars.[21]

Subgroup analysis

The heterogeneity may come from such factors as large differences in the years of publication, different population backgrounds, and inconsistent acceptance criteria for patients. First of all, we will preliminarily evaluate the reliability of the meta-analysis results through sensitivity analysis (excluding some low-quality studies). Then, on this basis, we will also conduct subgroup analysis to compare the efficacy of each subgroup, so as to determine whether different regions, races and other factors may affect the research results.

Statistical analyses

In previous Meta-analysis publication[22] we used ADDIS software, it will be different next. We plan to use STATA16.0 software to draw a network diagram of the comparison of various interventions, and use Markov Chain Monte Carlo (MC-MC) method to simulate, the number of iterations is set to 50,000.[23] Interstudy heterogeneity will be evaluated by the Q statistic, where $P<0.10$ will be considered statistically significant and informative by I^2 statistic, where $I^2 \geq 50$ % will indicate heterogeneity. We will perform subgroup meta-analysis to assess differences.[24] In order to evaluate whether publication bias exists in the whole network, this study intends to use comparison-correction funnel plot[25]. The league table will be calculated for each main outcome endpoint, and the ranking results are reflected by the area under the cumulative ranking curve (SUCRA) [26]. To sum up, we will use the following two kinds of software for analysis at the same time, and the whole process will be checked by statistical experts. The general steps are as follows: first, we will make a network diagram and some forest diagrams according to the preprocessed data, and then, we will draw some ranking charts (net-league tables) for the efficacy comparison of each treatment method in strict accordance with the operating specifications. For each endpoint that meets the inconsistency test model, we will actively look for the source of heterogeneity, and conduct sensitivity analysis and subgroup analysis, eventually give a reasonable explanation for the results. Finally, we would cumulative probability of all observed endpoints and rank these treatments from priority to inferiority in tabular forms.

For peer review only - http://bmjopen.bmj.com/site/about/guidelines.xhtml

For peer review only

The software to be used in this study are STATA 16.0 (Stata Corporation, College Station, TX 77845 USA) and Review Manager 5.4 (Oracle Corporation, The Cochrane Collaboration, 2020).

DISSCUSSION

As mentioned above, although the ONCAB or CECC has always been the gold standard for the treatment of LMCA diseases, with the rise of minimally invasive surgery, the discussion about the best strategy for revascularization of left main artery lesions is controversial in clinic.[2] Although numerous RCTs have compared CABG with PCI, no studies have been powered to detect a difference in mortality during the long follow-up among them. One study[27] has reported that no benefit for CABG over PCI was seen in patients with left main disease (CABG had a mortality benefit over PCI in patients with multivessel disease, and those with diabetes and higher coronary complexity.).

Although the current clinical guidelines have pointed that the SYNTAX score could help select the vascular reconstruction strategy for unprotected left main disease(ULMC)[28], one study of ten-year outcomes has shown that the discriminative capacity of SYNTAX score was relevant in the PCI group but not in the CABG group.[29]

Previous meta-analysis[9-13] showed that compared with traditional coronary artery bypass grafting, different surgical methods had certain advantages in different indicators. However, for the newly developed surgical treatment methods in recent years, such as robotic coronary artery bypass grafting[11], the number of randomized controlled trials is limited and lacks convincing, and there is no systematic and comprehensive comparison.

In order to ensure the quality of research, the authors will follow strict guidelines in the review process and their reports, such as PRISMA-P and PRISMA-ScR.[30] In order to avoid possible methodological defects, we will use the latest guideline provided by The Joanna Briggs Institute (JBI) in 2020 when conducting the scope review.[31] Our proposed program was registered in a predefined manner to increase the transparency and reliability of the review results.[32]

Of course, our research also has limitations. For example, although there are extensive search strategies, we only include literature with English language. Others may worry that there are confounding factors, such as different surgeons experience, population baseline characteristics, which may cause the different results of the entire study. However, as long as enough randomized controlled studies that meet the eligibility criteria are included, the stable results of the network analysis of large samples will mask this effect. In addition, when indirect comparison cannot be conducted in any case, we will conduct reliable direct comparison analysis results. If quantitative synthesis is not appropriate, narrative synthesis will be used.

In summary, the study planned by our team may be a relatively comprehensive and authentic comparison in the treatments about left main coronary artery disease. The analysis results will be used to provide some decision-making help for the best choice of Which coronary artery bypass grafting strategy or PCI.

Patient and public involvement

As the proposed systematic review will be conducted based on published studies, no patients and members of the public will be directly involved.

Amendments

Any amendments to this protocol will be documented.

Planned start and end date

For peer review only - http://bmjopen.bmj.com/site/about/guidelines.xhtml

The review is planned to start on 1 November 2021 and end on 1 June 2023.

Ethics and dissemination

The essence of this study is to summarize and analyze the original data without the approval of the ethics committee. Our research does not involve ethical issues, and the results will be published in peer review journals.

Contributors WMH: Concept research methodology, database search, article screening, data extraction, quality evaluation and drafting. BH: Database search, article screening and data extraction will be conducted. QL: Make the screening form, and judge the inconsistent opinions. MLC: Literature quality evaluation and statistical analysis. LW: Participate in the outcome discussion. All authors will read and approve the final manuscript.

Funding Not applicable.

Competing interests None declared.

Patient consent for publication Not applicable.

Provenance and peer review Not commissioned; external peer review

Open access This is an open access article distributed in accordance with the Creative Commons Attribution non-Commercial (CC BY- NC 4.0) license, which permits others to distribute, remix, adapt, build upon this work non- commercially, and license their derivative works on different terms, provided the original work is properly cited, appropriate credit is given, any changes made indicated, and the use is non- commercial. See: http://creativecommons. org/ licenses/ by-nc/ 4. 0/.

ORCID iDs

Weimin Huang http://orcid.org/0000-0003-2556-6214

For peer review only

REFERENCES

1. Holm NR, Makikallio T, Lindsay MM, et al. Percutaneous coronary angioplasty versus coronary artery bypass grafting in the treatment of unprotected left main stenosis: updated 5-year outcomes from the randomised, non-inferiority NOBLE trial. *Lancet* 2020;395(10219):191-99. doi: 10.1016/S0140-6736(19)32972-1 [published Online First: 2019/12/28]

2. Alasnag M, Yaqoub L, Saati A, et al. Left Main Coronary Artery Interventions. *Interv Cardiol* 2019;14(3):124-30. doi: 10.15420/icr.2019.10.R2 [published Online First: 2019/12/25]

3. Daemen J, Boersma E, Flather M, et al. Long-term safety and efficacy of percutaneous coronary intervention with stenting and coronary artery bypass surgery for multivessel coronary artery disease: a meta-analysis with 5-year patient-level data from the ARTS, ERACI-II, MASS-II, and SoS trials. *Circulation* 2008;118(11):1146-54. doi: 10.1161/CIRCULATIONAHA.107.752147 [published Online First: 2008/08/30]

4. Doucet S, Jolicoeur EM, Serruys PW, et al. Outcomes of left main revascularization in patients with acute coronary syndromes and stable ischemic heart disease: Analysis from the EXCEL trial. *Am Heart J* 2019;214:9-17. doi: 10.1016/j.ahj.2019.04.016 [published Online First: 2019/06/01]

5. Stone GW, Kappetein AP, Sabik JF, et al. Five-Year Outcomes after PCI or CABG for Left Main Coronary Disease. *N Engl J Med* 2019;381(19):1820-30. doi: 10.1056/NEJMoa1909406 [published Online First: 2019/09/29]

6. Marui A, Okabayashi H, Komiya T, et al. Benefits of off-pump coronary artery bypass grafting in high-risk patients. *Circulation* 2012;126(11 Suppl 1):S151-7. doi: 10.1161/CIRCULATIONAHA.111.083873 [published Online First: 2012/11/06]

7. Zubarevich A, Kadyraliev B, Arutyunyan V, et al. On-pump versus off-pump coronary artery bypass surgery for multi-vessel coronary revascularization. *J Thorac Dis* 2020;12(10):5639-46. doi: 10.21037/jtd-20-1284 [published Online First: 2020/11/20]

8. Shroyer AL, Grover FL, Hattler B, et al. On-pump versus off-pump coronary-artery bypass surgery. *N Engl J Med* 2009;361(19):1827-37. doi: 10.1056/NEJMoa0902905 [published Online First: 2009/11/06]

9. Kowalewski M, Pawliszak W, Raffa GM, et al. Safety and efficacy of miniaturized extracorporeal circulation when compared with off-pump and conventional coronary artery bypass grafting: evidence synthesis from a comprehensive Bayesian-framework network meta-analysis of 134 randomized controlled trials involving 22 778 patients. *European journal of cardio-thoracic surgery : official journal of the European Association for Cardio-thoracic Surgery* 2016;49(5):1428-40. doi: 10.1093/ejcts/ezv387 [published Online First: 2015/11/06]

10. Indja B, Woldendorp K, Black D, et al. Minimally invasive surgical approaches to left main and left anterior descending coronary artery revascularization are superior compared to first- and second-generation drug-eluting stents: a network meta-analysis. *European journal of cardio-thoracic surgery : official journal of the European Association for Cardio-thoracic Surgery* 2020;57(1):18-27. doi: 10.1093/ejcts/ezz184 [published Online First: 2019/06/21]

11. Hammal F, Nagase F, Menon D, et al. Robot-assisted coronary artery bypass surgery: a systematic review and meta-analysis of comparative studies. *Canadian journal of surgery Journal canadien de chirurgie* 2020;63(6):E491-E508. [published Online First: 2020/11/07]

12. Leonard JR, Rahouma M, Abouarab AA, et al. Totally endoscopic coronary artery bypass surgery: A meta-analysis of the current evidence. *Int J Cardiol* 2018;261:42-46. doi: 10.1016/j.ijcard.2017.12.071 [published Online First: 2018/04/17]

13. Reynolds AC, King N. Hybrid coronary revascularization versus conventional coronary artery bypass grafting: Systematic review and meta-analysis. *Medicine (Baltimore)* 2018;97(33):e11941. doi: 10.1097/MD.0000000000011941 [published Online First: 2018/08/17]

14. Kayatta MO, Halkos ME. Reviewing hybrid coronary revascularization: challenges, controversies and opportunities. *Expert Rev Cardiovasc Ther* 2016;14(7):821-30. doi: 10.1080/14779072.2016.1174576 [published Online First: 2016/04/05]

15. Zhou S, Fang Z, Xiong H, et al. Effect of one-stop hybrid coronary revascularization on postoperative renal function and bleeding: a comparison study with off-pump coronary artery bypass grafting surgery. *The Journal of thoracic and cardiovascular surgery* 2014;147(5):1511-16 e1. doi: 10.1016/j.jtcvs.2013.05.026 [published Online First: 2013/07/25]

16. Shamseer L, Moher D, Clarke M, et al. Preferred reporting items for systematic review and meta-analysis protocols (PRISMA-P) 2015: elaboration and explanation. *BMJ* 2015;350:g7647. doi: 10.1136/bmj.g7647 [published Online First: 2015/01/04]

17. Moher D, Shamseer L, Clarke M, et al. Preferred reporting items for systematic review and meta-analysis protocols (PRISMA-P) 2015 statement. *Syst Rev* 2015;4:1. doi: 10.1186/2046-4053-4-1 [published Online First: 2015/01/03]

18. Higgins JP, Altman DG, Gotzsche PC, et al. The Cochrane Collaboration's tool for assessing risk of bias in randomised trials. *BMJ* 2011;343:d5928. doi: 10.1136/bmj.d5928 [published Online First: 2011/10/20]

19. Page MJ, McKenzie JE, Bossuyt PM, et al. The PRISMA 2020 statement: an updated guideline for reporting systematic reviews. *BMJ* 2021;372:n71. doi: 10.1136/bmj.n71 [published Online First: 2021/03/31]

20. Levac D, Colquhoun H, O'Brien KK. Scoping studies: advancing the methodology. *Implement Sci* 2010;5:69. doi: 10.1186/1748-5908-5-69 [published Online First: 2010/09/22]

21. Guyatt G, Oxman AD, Akl EA, et al. GRADE guidelines: 1. Introduction-GRADE evidence profiles and summary of findings tables. *J Clin Epidemiol* 2011;64(4):383-94. doi: 10.1016/j.jclinepi.2010.04.026 [published Online First: 2011/01/05]

22. Huang W, Hou B, Li Q, et al. Comparative efficacy of five surgical methods in the treatment of mitral regurgitation: A systematic review and network meta-analysis. *J Card Surg* 2021 doi: 10.1111/jocs.16085 [published Online First: 2021/10/19]

23. Wang J, Zhu X, Sun Y, et al. Efficacy and safety of traditional Chinese medicine combined with routine western medicine for the asymptomatic novel coronavirus disease (COVID-19): A Bayesian network meta-analysis protocol. *Medicine (Baltimore)* 2020;99(35):e21927. doi: 10.1097/MD.0000000000021927 [published Online First: 2020/09/03]

24. Brignardello-Petersen R, Izcovich A, Rochwerg B, et al. GRADE approach to drawing conclusions from a network meta-analysis using a partially contextualised framework. *BMJ* 2020;371:m3907. doi: 10.1136/bmj.m3907 [published Online First: 2020/11/12]

25. Shim S, Yoon BH, Shin IS, et al. Network meta-analysis: application and practice using Stata. *Epidemiol Health* 2017;39:e2017047. doi: 10.4178/epih.e2017047 [published Online First: 2017/11/03]

26. Kuo FC, Hsu CW, Tan TL, et al. Effectiveness of Different Wound Dressings in the Reduction of Blisters and Periprosthetic Joint Infection After Total Joint Arthroplasty: A Systematic Review and Network Meta-Analysis. *J Arthroplasty* 2021;36(7):2612-29. doi: 10.1016/j.arth.2021.02.047 [published Online First: 2021/03/13]

For peer review only - http://bmjopen.bmj.com/site/about/guidelines.xhtml

27. Head SJ, Milojevic M, Daemen J, et al. Mortality after coronary artery bypass grafting versus percutaneous coronary intervention with stenting for coronary artery disease: a pooled analysis of individual patient data. *Lancet* 2018;391(10124):939-48. doi: 10.1016/S0140-6736(18)30423-9 [published Online First: 2018/02/27]

28. Freemantle N, Pagano D. Concerns with the new SYNTAX score. *Lancet* 2021;397(10276):795. doi: 10.1016/S0140-6736(21)00223-3 [published Online First: 2021/03/01]

29. Yoon YH, Ahn JM, Kang DY, et al. Impact of SYNTAX Score on 10-Year Outcomes After Revascularization for Left Main Coronary Artery Disease. *JACC Cardiovasc Interv* 2020;13(3):361-71. doi: 10.1016/j.jcin.2019.10.020 [published Online First: 2020/02/08]

30. Tricco AC, Lillie E, Zarin W, et al. PRISMA Extension for Scoping Reviews (PRISMA-ScR): Checklist and Explanation. *Ann Intern Med* 2018;169(7):467-73. doi: 10.7326/M18-0850 [published Online First: 2018/09/05]

31. Peters MDJ, Marnie C, Tricco AC, et al. Updated methodological guidance for the conduct of scoping reviews. *JBI Evid Synth* 2020;18(10):2119-26. doi: 10.11124/JBIES-20-00167 [published Online First: 2020/10/11]

32. Page MJ, Shamseer L, Tricco AC. Registration of systematic reviews in PROSPERO: 30,000 records and counting. *Syst Rev* 2018;7(1):32. doi: 10.1186/s13643-018-0699-4 [published Online First: 2018/02/22]

For peer review only

For peer review only - http://bmjopen.bmj.com/site/about/guidelines.xhtml

Appendix: Search Strategies

PubMed

#1"Coronary Disease"[Mesh] or "Left Main Disease*"[tw] or "Coronary Arteriosclerosis*"[tw]
#2"Coronary Artery Bypass"[Mesh] or "Myocardial Revascularization"[Mesh] or "Angioplasty, Balloon, Coronary"[Mesh] or "CABG"[tw]
#3 #1 OR #2
#4 "Surgical Procedures, Operative"[Mesh] or "Off-Pump Coronary Artery Bypass" [tw] or "Off-Pump Coronary Artery Bypass"[tw]
#5"Percutaneous Coronary Intervention"[Mesh] or "Robotic Surgical Procedures"[Mesh] or "Video-Assisted Surgery"[Mesh] or "Thoracoscopes"[Mesh] or "Thoracotomy"[Mesh]
#6 "Traditional thoracotomy"[tw] or "Conventional Surgery"[tw] or "Hybrid"[tw]
#7 #4 or #5 or #6
#8"random*"[tw] or "controlled"[tw] or "trial*"[tw] or "groups"[tw]
#9(("singl*"[tw] or "doubl*"[tw] or "tripl*"[tw]) and ("mask*"[tw] or "blind*"[tw]))
#10 #8 or #9
#11 #3 and #7 AND #10

Embase (Elsevier)

#1 ‘Coronary Disease’/exp
#2 ‘Coronary Artery Disease’/exp
#3 (“Left Main Diseases” or “Coronary Arteriosclerosis” or CABG): ti,ab
#4 #1 or #2 or #3
#5 ‘surgery’/exp
#6 “Operative Procedure”: ti,ab
#7 #5 or #6
#8‘Percutaneous Coronary Intervention’/exp
#9 “Percutaneous Coronary Revascularizations”: ti,ab
#10 #8 or #9
#11 ‘Robotic Surgical Procedures’/exp
#12 (“Robotic-Assisted Surgery” or “Robot Surgery”): ti,ab
#13 #11 or #12
#14“Video Assisted Surgery”: ti,ab
#15 ‘Video-Assisted Surgery’/exp
#16 #14 or #15
#17 ‘Thoracoscopes’/exp
#18 (“Pleuroscop*” or Thoracoscopy or “Endoscop*”): ti,ab

For peer review only

#19 #17 or #18
#20 'Thoracotomy'/exp
#21 ("Sternotom*" or "thoracotom*" or "hybrid"): ti,ab
#22 #20 or #21
#23 #7 or #10 or #13 or #16 or #19 or #22
#24 ("random*" or "control*" or "trial*" or placebo): ti,ab
#25 (("singl*" or "doubl*" or "tripl*") and ("mask*" or "blind*")): ti,ab
#26 #23 or #24
#27 #4 AND #23 AND #26

Web of Sience

#1 "Coronary Artery Disease" or "Coronary Disease"
#2 "Left Main Disease*" or "Coronary Arteriosclerosis*"
#3 "Coronary Artery Bypass" or "Coronary Artery Bypass, Off Pump" or "Myocardial Revascularization"
#4 "Off-Pump Coronary Artery Bypass" or "Beating Heart Coronary Artery Bypass"
#5 #1 or #2 or #3 or #4
#6 "Surgical Procedures, Operative" or "Operative Surgical Procedure"
#7"Percutaneous Coronary Intervention" or "Percutaneous Coronary Revascularizations"
#8" Robot Surger*" or "Robotic-Assisted Surger*" or "Robotic Surgical Procedures"
#9" Video-Assisted Surger*" or "Video Assisted Surger*"
#10 "Thoracoscop*" or "Pleuroscope*" or "Endoscop*"
#11 "Thoracotom*" or "Thoracic Surgery" or "Sternotom*"
#12 #6 or #7 or #8 or #9 or #10 or #11
#13 #5 and #12

The Cochrane Library (Wiley Online Library)

#1 MeSH descriptor 'Coronary Disease' explode all trees
#2 ("Left Main Diseases" or "Coronary Arteriosclerosis"): ti,ab,kw
#3 MeSH descriptor 'Coronary Artery Bypass' explode all trees
#4 ("Off-Pump Coronary Artery Bypass" or "Beating Heart Coronary Artery Bypass"): ti,ab,kw
#5 #1 or #2 or #3 or #4
#6 MeSH descriptor 'Operative Surgical Procedure' explode all trees
#7 ("Operative Procedure*" or "Surgery, Ghost" or Surgery): ti,ab,kw
#9 MeSH descriptor' Percutaneous Coronary Intervention' explode all trees
#10 "Percutaneous Coronary Revascularizations": ti,ab
#12 MeSH descriptor 'Robotic Surgical Procedures' explode all trees
#13 ("Robot Surger*" or "Robotic-Assisted Surgery*"): ti,ab
#14 MeSH descriptor 'Video-Assisted Surgery' explode all trees

For peer review only

#15 (“Video Assisted Surgery”: or Thoracoscopes): ti,ab,kw:
#16MeSH descriptor ‘Sternotomy’ explode all trees
#17 “Traditional thoracotomy” or “Median thoracotomy”: ti,ab,kw:
#18 #6 or #7 or #8 or #9 or #10 or #11 or #12 or #13 or #14 or #15 or #16 or #17
#19 #5 and #18 in Trials

For peer review only

PRISMA-P (Preferred Reporting Items for Systematic review and Meta-Analysis Protocols) 2015 checklist: recommended items to address in a systematic review protocol*

Section and topic	Item No	Checklist item
**ADMINISTRATIVE INFORMATION**		
Title:		
Identification	1a	Comparative efficacy of 8 therapeutic methods in the treatment of left main coronary artery disease: a Bayesian network meta-analysis protocol (Page 1 line 1-2)
Update	1b	None
Registration	2	PROSPERO registration number CRD42021274712(Page 1 line 33)
Authors:		
Contact	3a	All names, institutional affiliations, e-mail address of all protocol authors are provided as well as physical mailing address of corresponding author.
Contributions	3b	The contributions of protocol authors are listed and the guarantor of the review is identified (Page 5 line 43-47)
Amendments	4	Amendments are not expected but all deviations will be documented and discussed (Page 5 line 31-32)
Support:		
Sources	5a	Not applicable. (Page 5, line 14)
Sponsor	5b	Individual: Huang Weimin, the researcher of the team. (Page 1, 5)
Role of sponsor or funder	5c	The sponsor is the one of researchers in the team, who played the role of a correspondent and made a significant contribution to the program. (Page 5)
**INTRODUCTION**		
Rationale	6	The rationale for the review is described in contrast to what is already known and the gaps in literature (Page 2-3)
Objectives	7	We provided our explicit objectives (Page 1) and the participants, interventions, comparators, and outcomes (PICO) on (Page 2-3)
**METHODS**		
Eligibility criteria	8	We explicitly described our inclusion and exclusion criteria (Page 3). (such as PICO, study design, setting, time frame) and report characteristics (such as years considered, language, publication status) to be used as criteria for eligibility for the review (Page 3 line 4)
Information sources	9	We described our search strategy, databases that will be used and data sources (Page 2)
Search strategy	10	The search strategy to be used for 4 electronic databases, including planned limits, such that it could be repeated. (Page 2)
Study records:		
Data management	11a	Endnote software was used to classify and arrange the searched literature according to the surgical method (Page3 line 37)

For peer review only

Selection process	11b	We clearly state the process that will be used for selecting studies (Page 3 42-46)
Data collection process	11c	We described the plan of extracting data from reports (Page 4)
Data items	12	We listed and defined all variables for which data will be sought (Page 3)
Outcomes and prioritization	13	We listed and defined all outcomes for which data will be sought, including prioritization of main and additional outcomes, with rationale (Page 2, 4)
Risk of bias in individual studies	14	We described anticipated methods for assessing risk of bias of individual studies, including whether this will be done at the outcome or study level. We may use the Review Manager software. (Page 4)
Data synthesis	15a	We described criteria under which study data will be quantitatively synthesized (Page 3)
	15b	We described our plan to assess heterogeneity (Page 4 line33)
	15c	We describe our additional analyses (including sensitivity, subgroup analyses, and meta-regression) (Page 4 22-27)
	15d	If quantitative synthesis is not appropriate, narrative synthesis will be used. (Page 5 line 34)
Meta-bias(es)	16	We described the meta-bias (Page 4)
Confidence in cumulative evidence	17	We will use a quality score as described. (Page 4 line 15-20)

*** It is strongly recommended that this checklist be read in conjunction with the PRISMA-P Explanation and Elaboration (cite when available) for important clarification on the items. Amendments to a review protocol should be tracked and dated. The copyright for PRISMA-P (including checklist) is held by the PRISMA-P Group and is distributed under a Creative Commons Attribution Licence 4.0.**

From: Shamseer L, Moher D, Clarke M, Ghersi D, Liberati A, Petticrew M, Shekelle P, Stewart L, PRISMA-P Group. Preferred reporting items for systematic review and meta-analysis protocols (PRISMA-P) 2015: elaboration and explanation. BMJ. 2015 Jan 2;349(jan02 1):g7647.

For peer review only

PRISMA 2009 Flow Diagram

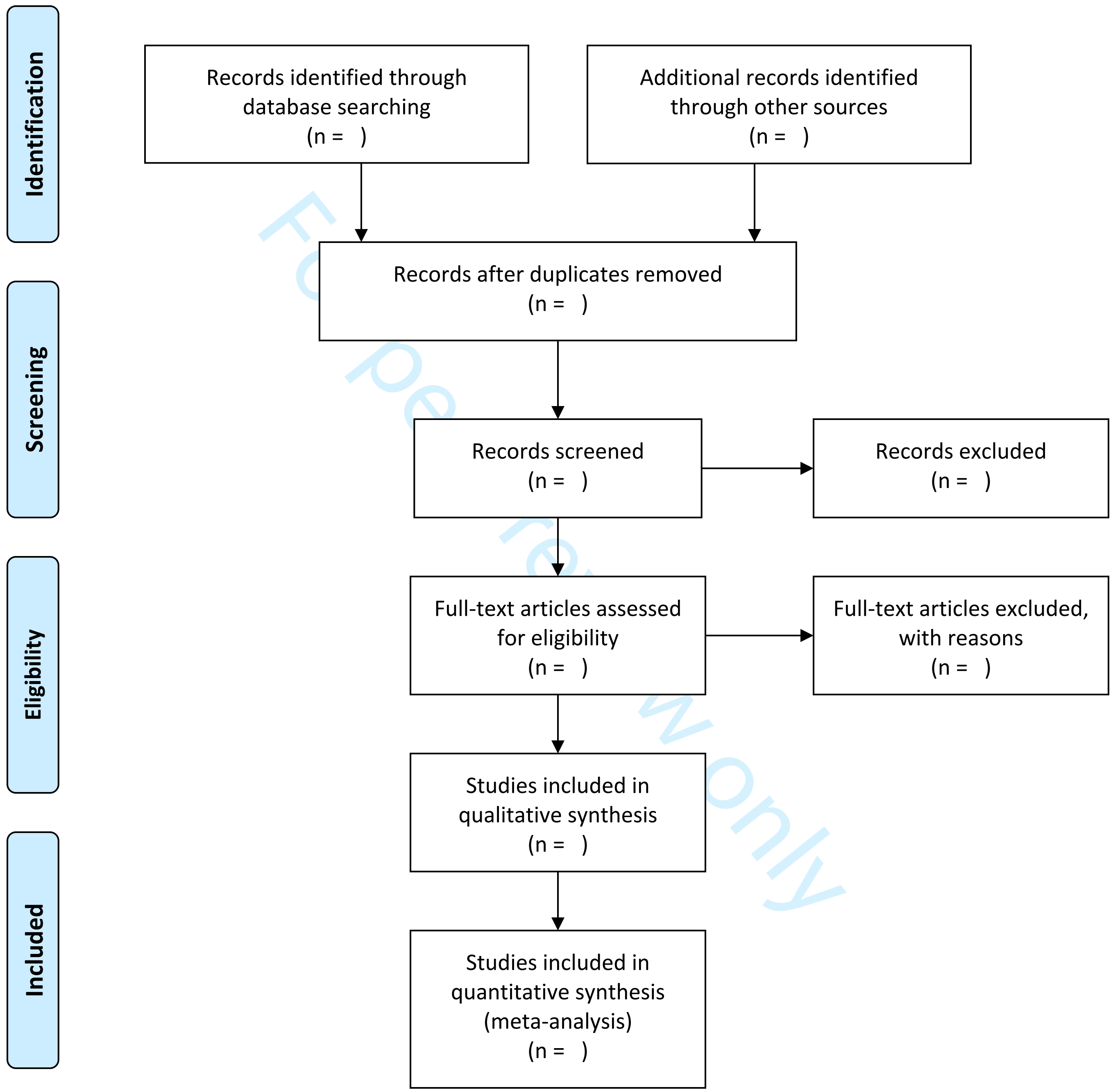

From: Moher D, Liberati A, Tetzlaff J, Altman DG, The PRISMA Group (2009). *P*referred *R*eporting *I*tems for *S*ystematic Reviews and *M*eta-*A*nalyses: The PRISMA Statement. PLoS Med 6(7): e1000097. doi:10.1371/journal.pmed1000097

For more information, visit www.prisma-statement.org.

For peer review only - http://bmjopen.bmj.com/site/about/guidelines.xhtml
